# Epigenetic Lens to Visualize the Severe Acute Respiratory Syndrome Coronavirus-2 (SARS-CoV-2) Infection in COVID-19 Pandemic

**DOI:** 10.3389/fgene.2021.581726

**Published:** 2021-03-22

**Authors:** Nitin Saksena, Srinivasa Reddy Bonam, Monica Miranda-Saksena

**Affiliations:** ^1^EPIGENES Australia Pty Ltd, Melbourne, VIC, Australia; ^2^Institute of Health and Sport, Victoria University, Footscray, VIC, Australia; ^3^Institut National de la Santé et de la Recherche Médicale, Centre de Recherche des Cordeliers, Equipe- Immuno-pathologie et Immuno-intervention Thérapeutique, Sorbonne Université, Université de Paris, Paris, France; ^4^Herpes Neuropathogenesis Research Group, The Westmead Institute for Medical Research, The University of Sydney, Sydney, NSW, Australia

**Keywords:** COVID, SARSCOV2, epigenetics, methylation, miRNA, chromatin, SARSCoV2 pathogenesis, epigenetic modulation

## Abstract

In <20 years, we have witnessed three different epidemics with coronaviruses, SARS-CoV, MERS-CoV, and SARS-CoV-2 in human populations, causing widespread mortality. SARS-CoV-2, through its rapid global spread, has led to the pandemic that we call COVID-19. As of February 1, 2021, the global infections linked to SARS-CoV-2 stand at 103,503,340, with 2,236,960 deaths, and 75,108,099 recoveries. This review attempts to highlight host-pathogen interaction with particular emphasis on the role of epigenetic machinery in regulating the disease. Although researchers, since the start of the pandemic, have been intensely engaged in diverse areas to understand the mechanisms involved in SARS-CoV-2 infection to find answers that can bring about innovative ways to swiftly treat and prevent disease progression, this review provides an overview on how the host epigenetics is modulated and subverted by SARS-CoV-2 to enter the host cells and drive immunopathogenesis. Epigenetics is the study that combines genetic and non-genetic factors controlling phenotypic variation, which are primarily a consequence of external and environmental stimuli. These stimuli alter the activity of a gene without impinging on the DNA code. In viral-host interactions, DNA/RNA methylation, non-coding RNAs, chromatin remodeling, and histone modifications are known to regulate and modulate host gene expression patterns. Viruses such as Coronaviruses (an RNA virus) show intrinsic association with these processes. They have evolved the ability to tamper with host epigenetic machinery to interfere with immune sensing pathways to evade host immune response, thereby enhancing its replication and pathogenesis post-entry. These epigenetic alterations allow the virus to weaken the host's immune response to successfully spread infection. How this occurs, and what epigenetic mechanisms are altered is poorly understood both for coronaviruses and other respiratory RNA viruses. The review highlights several cutting-edge aspects of epigenetic work primarily pertinent to SARS-CoV-2, which has been published between 2019 and 2020 to showcase the current knowledge both in terms of success and failures and take lessons that will assist us in understanding the disease to develop better treatments suited to kill SARS-CoV-2.

## Introduction

On December 31, 2019, the World Health Organization (WHO) was alerted by the China Health Authority (CHA) that several cases of pneumonia of unknown etiology were affecting the Wuhan City in Hubei Province in Central China (WHO, 2019). The new etiology was named Severe Acute Respiratory Syndrome (SARS)-associated coronavirus, SARS-CoV-2, and a new pandemic, called COVID-19 was born and-the third epidemic since SARS-CoV and MERS that appeared in 2002 and 2012, respectively. It is not uncommon, and there are human coronaviruses known to cause cough or a cold every flu season and go unnoticed because they don't cause serious respiratory complications. In contrast, the new SARS-CoV-2, which is the cause of the COVID-19 pandemic, has been defined by more severe respiratory illness and subsequent deaths globally. This pandemic has spread unabatedly despite quarantine measures attributed to undetectable or silent infection in asymptomatic carriers- a feature that has led to the rapid global spread of SARS-CoV-2 (PAHO, 2020; https://www.paho.org/en).

The last pandemic humans witnessed was that of Spanish Flu that occurred in December 1918 (Centers for Disease Control and Prevention, 2019), and it lasted until December 1920 infecting >500 million people, with an estimated death toll in the range of 50–100 million people, making it one of the deadliest pandemics in human history (Rosenwald, 2020).

As of February 1, 2021, the global infections linked to SARS-CoV-2 stand at 103,503,340, with 2,236,968 deaths, and 75,108,099 recoveries (PAHO, 2020, https://www.paho.org/en). An important fact to be noted is that the recovery rate is at 71%, and this recovery pattern varies and is dependent on demographics (PAHO, 2020). The death rate is at 2.1% as per global statistics and has also varied between demographies. As we have witnessed, the United States, Spain, Italy, and the United Kingdom bore the brunt of COVID-19 infections and related mortality in the earlier part of the pandemic, which also continues during the second wave of the pandemic. In contrast, Brazil and India along have experienced high infection rate with lower mortality rates than the aforementioned countries. The new rise in infection cases and related mortality in the US and Europe during the second wave is particularly concerning and could be attributed to the emergence of novel mutated variants of SARS-CoV-2, which remains to be confirmed, but it is being suggested that these mutations could provide virus an edge in transmissibility and renewed infectious potential (WHO, 2019; PAHO, 2020; European Centre for Disease Prevention and Control, 2021). Among the developed countries, Taiwan, Australia, and New Zealand have recorded the smallest number of cases and deaths compared to other geographical areas of the world (PAHO, 2020), largely attributed to effective control measures on human movements, rigorous testing and good surveillance and monitoring.

Among the list of approved treatments for SARS-CoV-2 are only the fast-tracked vaccines that are being currently administered globally (Prüβ, 2021), and for which long-term efficacy data remains to be proven in human patients. No antiviral drug has been proven to be completely effective to date. Noteworthy is that most drugs that were touted to be useful have not been created keeping SARS-CoV-2 in mind, but are being repurposed. As a consequence, there is an unmet and urgent need for effective therapeutics, specifically against the SARS-CoV-2 (PAHO, 2020). Current vaccines, if proven efficacious in the long-term, will pave the way for a durable outcome in the future. We have learned profound lessons from the previous outbreaks of the SARS-CoV, and MERS-Co-V and the associated mortality. This, in some ways, has acted as the food for thought in tackling the current pandemic, and probably will continue to help to strategize the future framework on treatments for SARS-CoV-2. The basic understanding of the biology of SARS-CoV-2 is being extrapolated from this prior knowledge, which will enlighten us on some of the unknown aspects of viral pathogenesis, which will allow the current drugs to have a chance of success in specifically treating SARS-CoV-2 (Gorbalenya et al., 2020). Although a record number of publications have appeared on SARS-CoV-2 between December 2019 and January 2021, this review attempts to showcase this current knowledge with particular emphasis on epigenetic regulation of the SARS-CoV-2, which has been less explored and discussed in the plethora of publications.

## Viral Transmission and Symptomatology of SARS-CoV-2 Infection

The most efficient way of viral transmission is through contact (person-to-person) via the droplets, and aerosol, which can travel some distance (PAHO, 2020). This dispersal of viral transmission is enhanced by population density (Li et al., 2020a). Intriguingly, it is apparent that the virus can be found in the feces, which can be another mode of viral transmission (Ghinai et al., 2020). Throughout the COVID-19 pandemic, we have learned that the biggest threat to viral transmission is posed by SARS-CoV-2-infected asymptomatic and pre-symptomatic carriers, in which the virus may or may not be detectable. The current evidence across diverse populations shows that asymptomatic and pre-symptomatic patients can test positive for SARS-CoV-2 at rates ranging from 17.9 to 57% with no symptoms (Lee et al., 2020). Thus, it is believed that the silent infection states may have a substantial contribution to the overall COVID-19 pandemic, and it has been suggested that the possible second wave or the new peaks of COVID-19 could be by these asymptomatic and pre-symptomatic carriers of the SARS-CoV-2 (Bai et al., 2020; Lai et al., 2020a). Further spread of the virus worldwide has been curtailed through quarantine and preventative measures that are in place globally. The use of masks, hand washing, and avoiding public contact has led to a considerable reduction in the transmission of the virus globally (PAHO, 2020).

Upon infection, most patients usually experience only mild to moderate symptoms with a high recovery rates. Symptoms vary, but the most common symptoms comprise of fever, dry cough, and tiredness; the less-common symptoms are aches and pains, sore throat, diarrhea, conjunctivitis, headache, loss of taste or smell, neurologic symptoms, a rash on the skin, or discoloration of fingers or toes. The severe symptoms, including difficulty in breathing or shortness of breath, chest pain or pressure, and loss of speech or movement (gov/coronavirus/2019-ncov/symptoms-testing/symptoms.html), and others. The serious breathing difficulty is a consequence of silent pneumonia that goes unnoticed. It suddenly culminates into severe pneumonia, also known as Acute Respiratory Distress Syndrome (ARDS), and is one of the significant features of SARS-CoV-2 infection and associated mortality especially in the elderly group of patients (Chen et al., 2020). The comorbidities (cardiovascular diseases, blood pressure, diabetes, chronic lung disease as a result of smoking, and immunosuppression as seen in cancer) predispose individuals to SARS-CoV-2 infection and lead to faster disease progression (Adhikari et al., 2020). On average, it takes 5–6 days post-infection with for symptoms to appear; however, it can take up to 14 days in many cases. There is a long list of currently defined symptoms resulting from SARS-CoV-2 infection, such as blood clot formation reminiscent of the catastrophic antiphospholipid syndrome, Kawasaki-like disease in children, neurological symptoms, and others. These symptoms vary between populations and also between individuals, thereby posing a real challenge in not only diagnosing the infection correctly but also in defining future treatment options for SARS-CoV-2 disease.

## Genomic Organization of SARS-CoV-2 and Clinical Characteristics

The SARS-CoV-2 is a positive-sense single-stranded enveloped RNA (+ssRNA) coronavirus (80–220 nm in diameter) that has led to the global pandemic started in Dec-2019. It belongs to the family Coronaviridae, consisting of Coronavirinae and Torovirinae sub-families (Gorbalenya et al., 2020).

**Figure 1:**
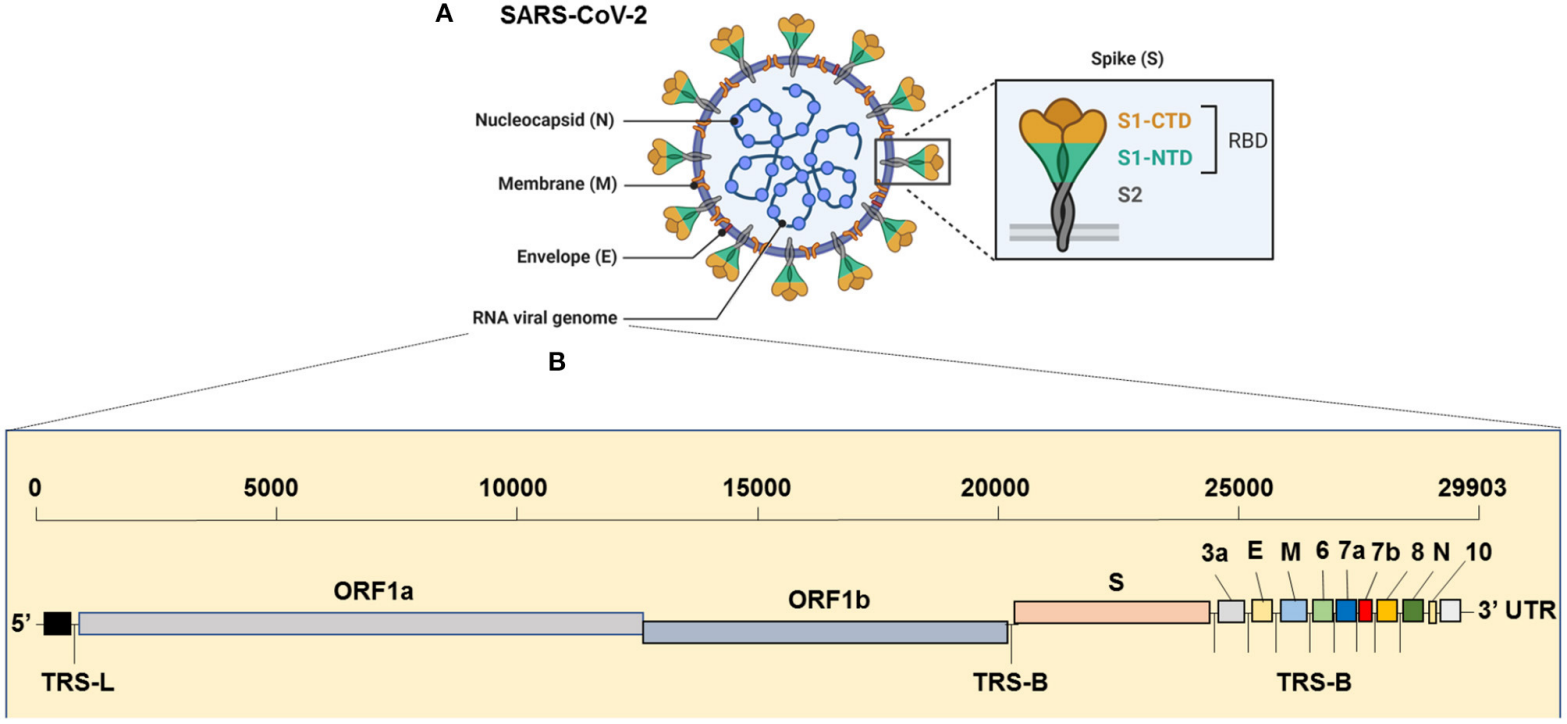
Genome Organization of SARS-CoV-2. **(A)** Complete virion structure: SARS-CoV-2 appears like a crown shape under the electron microscope. It is comprised with nucleocapsid (N), which wrapped the RNA genome, envelope (E), membrane (M), and spike (S) proteins. **(B)** RNA genome sequence (full-length RNA, 29,903 nucleotides (nt): SARS-CoV-2 composed with the total six open reading frames (ORFs) in which first two (ORF1a/b) occupy the two-third of the RNA genome. The later ORFs encode structural proteins, such as nucleocapsid (N), envelope (E), membrane (M), spike (S) and other accessory proteins (3a, 6, 7a, 7b, 8, and 10 [yet to validate its presence]). CTD, C-terminal domain; E, envelope; M, membrane; N, nucleocapsid; NTD, N-terminal domain; ORF1a/b, open reading frame; RBD, receptor-binding domain; SARS-CoV-2, Severe acute respiratory syndrome coronavirus 2; S, spike; TRS-B, transcription-regulatory sequence body; TRS-L, transcription-regulatory sequence leader.

**Figure 2:**
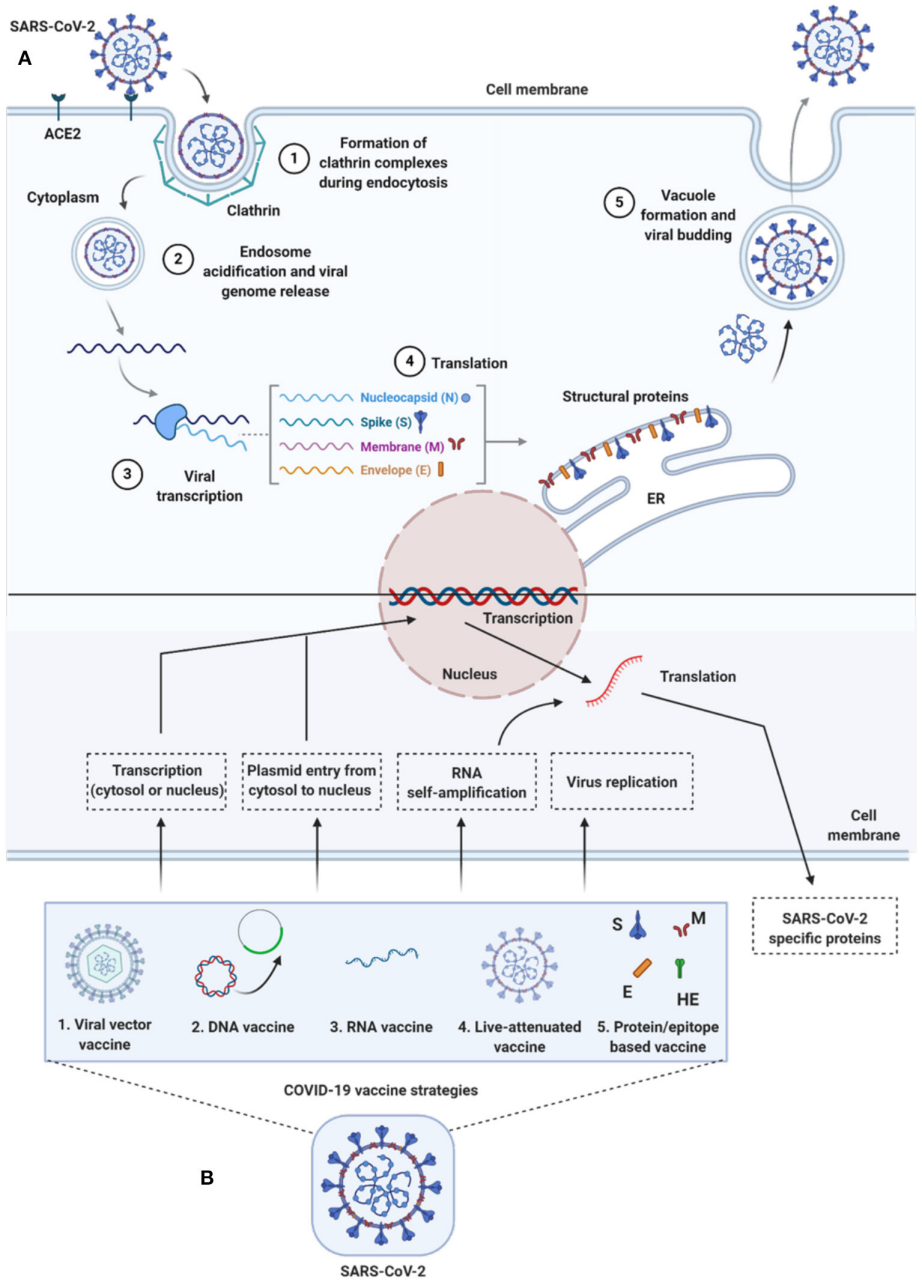
Life-cycle of SARS-CoV-2 and vaccine strategies for COVID-19. **(A)** Life-cycle of SARS-CoV-2 in host cells: Virus uses the ACE2 receptors (along with other co-receptors) for their internalization. It uses clathrin pits for endocytosis (1) and endosomes for genetic material release (2). Once RNA is released, it uses host cytoplasmic content for their transcription (3), translation (4), and vacuole formation and budding (5) followed by exocytosis. **(B)** Vaccine strategies against COVID-19: Diversified vaccine strategies have been proposed; viral vector vaccines (1), DNA vaccines (2), RNA vaccines (3), live-attenuated vaccines (4), and protein/epitope-based vaccines (5). ACE2, angiotensin I converting enzyme 2; DNA, deoxyribonucleic acid; E, envelope; ER, endoplasmic reticulum; HE, hemagglutinin-esterase; M, membrane; N, nucleocapsid; RNA, ribonucleic acid; S, spike; SARS-CoV-2, severe acute respiratory syndrome coronavirus 2.

The virus has the largest genome among all the known coronaviruses to date, comprising 29,903 bp in length (Khailany et al., 2020). The genome organization of the SARS-CoV-2 is 5′-RdRp-S-E-M-N-3′ and is highly conserved across coronaviruses (Brian and Baric, 2005). The 5′, which is more than two-thirds of the genome, is comprised of the open reading frame (ORF)1ab encoding ORF1ab polyproteins, whereas the one-third of the 3′ region consists of genes that encode structural proteins S (surface), E (Envelop), M (Membrane) and, N (nucleocapsid) proteins (Figure 1). Moreover, the SARS-CoV-2 codes for 6 accessory proteins, which are encoded by ORF3a, ORF6, ORF7a, ORF7b, and ORF8 genes (Figure 1). Structurally, the viral envelope is characterized by a crown-like shape (hence the name corona) bearing spikes, which are 20-nm in length as seen ultra-structurally (Goldsmith et al., 2004). The nucleoprotein (N) wraps the RNA genome and together forms a coiled tubular structure within the coronavirus particle, and this helical nucleocapsid is surrounded by the viral envelope (E), which also embeds the matrix protein (M). Like any other spike proteins seen across RNA viruses, SARS-CoV-2 also has the spike structural protein (S) that anchors the envelope, which plays a crucial role in virus attachment and fusion to host cell receptor. As a result, the spike protein is the main target of not only the neutralizing antibodies, but also of the drugs, such as fusion inhibitors and monoclonal antibodies, and several vaccine strategies that are in the pipeline or being trailed currently for SARS-CoV-2 globally (Figure 2). Several beta-coronaviruses possess the hemagglutinin esterase gene, in addition to Khailany et al. (2020) the five essential genes which code for four (N, E, M, S) structural proteins, and the RNA dependent RNA polymerase (RdRp), which is involved in viral replication and transcription (Figure 1). Thus, overall, the SARS-CoV-2 differs from SARS-CoV in their 3' open reading frames where SARS-CoV-2 possesses an Orf3b and Orf10 displaying low protein homology to SARS-CoV (Chan et al., 2020; Gordon et al., 2020). Besides, the Orf8 is intact in SARS-CoV-2, whereas Orf8a and b are encoded by SARS-CoV (Chan et al., 2020).

## Viral Entry, Host-Pathogen Interaction, and Pathogenesis

The first successful isolation of the SARS-CoV-2 was obtained from bronchoalveolar lavage of Chinese adult patients (Zhu et al., 2020), with subsequent detection of viral RNA in the throat and oral-and-nasopharyngeal swabs (saliva and nose), blood, urine, and stool (To et al., 2020a).

To enter host cells, like other viruses, coronaviruses also first bind to the host cell surface receptor, and upon attachment, the virus subsequently enters endosomes, leading to fusion with viral and host lysosomal membranes (To et al., 2020a) (Figure 2). To date, only preliminary studies have been done, suggesting that both SARS-CoV-2 and SARS-CoV use angiotensin-converting enzyme 2 (ACE2) to enter the host cell, which is attributed to similar receptor-binding protein structures between these two viruses. Comparative studies based on the crystal structure of the three coronaviruses- the SARS-CoV, SARS-CoV-2, and MERS-CoV, have revealed differences in binding avidity of the spike protein of SARS-CoV and SARS-CoV-2 to their cognate receptor ACE2 on the host cells, with MERS-CoV specifically binding to dipeptidyl peptidase (Shang et al., 2020; Zhang et al., 2020). One of the unique features of the SARS-CoV-2 is that its spike protein contains a proprotein convertase (PPC) motif at the S1/S2 junction. The PPC motif's role is unclear, and its cleavage from the spike protein has also failed to show any evidence of enhancement of viral entry into the host cells (Walls et al., 2020).

There are two distinctive domains in the receptor-binding S1 subunit of spike proteins in coronaviruses, containing the N-and-C-terminal domains (S1-NTD and S1-CTD), and both these domains engage in receptor binding activities and are the most common receptor binding domains (RBD) across coronaviruses. The RBD is among the regions with the highest genetic variability and also variability tolerance because of the many host selection pressures it is continuously exposed to. The S1 domain contains a receptor-binding domain (RBD) that uniquely recognizes and engages with the ACE2 for binding and entry. The RBD is flexible, and its constant switching from a standing-up position to a lying-down position aids in immune evasion from the host (Yuan et al., 2017). It is interesting to point out that the recent cryo-electron microscopy (cryo-EM) structure of the SARS-CoV-2 spike has demonstrated that the RBD is mostly in the lying-down position than upright (Wrapp et al., 2020), a state associated immune evasion. The fusion between host cell receptor ACE2 (hACE2), and virus particle is activated by proteolytic mechanisms in the host at the S1/S2 boundary, and as a consequence, S1 dissociates whereas the S2 undergoes a conformational change (Belouzard et al., 2012). It is important to note that the SARS-CoV-2 entry-activating proteases also engage cell surface protease Transmembrane Serine Protease 2 (TMPRSS2) and lysosomal proteases cathepsins for completing viral entry (Belouzard et al., 2012) (Figure 2) and that the serine protease TMPRSS2 is used for S protein priming (Hoffmann et al., 2020). Further, Hoffman et al. have shown that the treatment of the Calu-3 human lung cell line with the serine protease inhibitor Camostat mesylate partially blocked vesicular stomatitis virus (VSV) entry pseudo-types expressing the S protein of SARS-CoV-2 (Figure 2). Together, both ACE2 and TMPRSS2 play a vital role in SARS-CoV-2 entry into the host cell.

Overall, viral entry presents as a significant target for therapeutic intervention, and concerning SARS-CoV-2, a critical understanding of the receptor recognition mechanisms is sorely needed, which can provide vital insights into its receptor-binding affinity in the human host.

## Role of Epigenetics in SARS-CoV-2 Infection, Immune-Pathogenesis, and Comorbidities

### Epigenetic Regulation of Innate Host Immunity and Viral Pathogenesis

Epigenetic mechanisms clearly appear to be a vital part of SARS-CoV-2, which can make inroads into the host through these mechanisms resulting in the severity of illness and mortality that has been so frequently seen during the COVID-19 pandemic (Mehta et al., 2020). We know for many decades that invariably, all viruses use epigenetic mechanisms, especially CpG methylation, to induce enterocytosis and syncytium development- a critical feature of coronaviruses in general (Mehta et al., 2020; Xia et al., 2020). The underlying molecular and epigenetic mechanisms that regulate the pathogenesis of coronaviruses are complex and dependent on the host-virus interactions guiding viral entry, replication, and immuno-pathogenesis. SARS-CoV-2 is no different from other coronaviruses, as it also has an intrinsic ability to tamper with the host innate immune system. Although with subtle differences between DNA and RNA viruses, invariably, all viruses make use of host epigenetic reprogramming, which assists them in evading the host immune responses (Mehta et al., 2020).

To successfully survive and replicate through their life cycle in the host, viral pathogens have an arsenal of a variety of epigenetic strategies they use in subverting the host's immune system. These strategies include pathogen-directed modification of host proteins and chromatin by virus-specific gene products or viral proteins, modulation of activators and repressors in innate immunity that can attenuate pattern recognition receptor (PRR) sensing and signaling pathways. In response, the host immunity also counters pathogen-induced changes to their epigenomes to maintain effective control of anti-viral immunity. Thus, viruses have evolved diverse strategies to tamper with the host epigenetic machinery by targeting DNA methylation and reprogramming the host DNA methylome for its own benefit (Zhang and Cao, 2019).

While mutations can change the genetic code, thereby directly affecting the genetic material, epigenetic regulation, in contrast, bridges genotype and phenotype, therefore these modifications result in changes in the chromatin structure or modification of nucleic acid without any alteration in the genetic code. It implies the reversibility, flexibility, and quick responsiveness of epigenetic alterations to rapid changes in the environment and other exposures. Here, the environment and the genome serve as powerful interfaces for any epigenetic modification (Goldberg et al., 2007). Rapid progress has been made in many areas of medicine, which include cancer biology, infectious diseases, and immunity, and it is thought that some viral pathogens that modify chromatin and other epigenetic machinery, are excellent candidates for drug targets (Esteller, 2008; Obata et al., 2015).

The research has primarily focused on molecular mechanisms of RNA viruses that tamper with the components that regulate host innate immunity, which forms the anti-viral defense arsenal of the host. It has been suggested that the RNA viruses, especially that replicate in the cell cytoplasm, have evolved a sophisticated mechanism that is designed to not only to exert influence on the host epigenome but also regulate it, thereby taking charge of subverting the anti-viral defense of the host at its own terms to promote its own replication and successful establishment in the host (Bird, 2007; Goldberg et al., 2007). In support of this, it has been previously shown that both DNA and RNA viruses have evolved this function to antagonize the regulatory machinery of the host epigenome to facilitate viral replication and spread (Busslinger and Tarakhovsky, 2014).

Epigenetic mechanisms can switch genes on or off and determine which proteins need to be transcribed at any given time, thereby an essential role in regulating normal cellular processes. Different kinds of cells and organs have a different set of genes that turn on and off as per functional needs. Epigenetic silencing is one way to turn genes off, which contributes to differential expression, implying that such genomic changes that result in no sequence alteration in the host can be reversed in certain situations. Within these different cells that perform diverse functions, three systems act in tandem to silence genes, and this involves RNA-mediated silencing, DNA methylation, and histone modifications (Egger et al., 2004; Schäfer and Baric, 2017). These three systems, which are the functional regulators that modulate gene expression, work in tandem in a highly flexible and seamless manner.

Epigenetic modification of cellular genomes occurs in a highly structured and specific manner and is carefully orchestrated, particularly DNA methylation and other specific histone modifications that assure precise and reliable transmission of gene expression to the progeny cells (Schäfer and Baric, 2017). Upon infection and also during chronic infection, there is a disruption of cellular epigenetic balance (Schäfer and Baric, 2017), and it is this balance that defines the pathogenesis, in part, during infection, in addition to various disease processes in cancer, neurodegenerative and metabolic disorders. There is an intense research activity concerning SARS-CoV-2 on how this and the family of coronaviruses affect the host gene expression regulation. Although the complex epigenetic interactions of coronaviruses and epigenetic processes with host cells have been researched since the discovery of SARS virus in 2002 (Froude and Hughes, 2020), the current investigations are focussed on how the histone methylation/chromatin remodeling, DNA transcription, cellular packaging of DNA and non-coding RNAs epigenetically regulate gene expression (Schäfer and Baric, 2017). These processes are critical and vital for the virus as it relies on the host cell to replicate its genetic material and continue its progeny. There are four main aspects of epigenetic regulation (DNA methylation and oxidation, histone modifications/chromatin remodeling, and non-coding RNAs (miRNA), which are involved in shaping the innate immune response during any viral infection. Therefore, a clear understanding of these aspects can guide us in treating viral infections and various other diseases that afflict humans. High throughput genomic technologies are already allowing a comprehensive visualization and investigation of such epigenetic modifications with high resolution. With data integration being a reality, the possibilities are endless for a holistic view of these processes.

### DNA Methylation, Histone Modification, and Chromatin Remodeling

DNA methylation is a chemical process that adds a methyl group to DNA and is involved in transcriptional silencing. It is highly orderly and takes place post-DNA replication across all mammalian cells. It occurs explicitly at the 5′ position of the cytosine ring within the CpG islands, where a methyl group is added to create 5-methylcytosine (5 mC). This reaction is mediated by the DNA methyltransferases (DNMTs), which preferentially targets unmethylated CpG islands to achieve DNA methylation, and the insertion of methyl groups alters the structural appearance of DNA (Egger et al., 2004; Jones, 2012).

Through this epigenetic alteration, as stated earlier, viruses can switch on and switch off genes at multiple host gene locations. Such chemical modifications in DNA methylation and histone modification collectively subjugate the production of antigen presentation molecules vital in mounting anti-viral response during infection, as shown for both MERS-CoV and H5N1 viruses (Menachery et al., 2018). The two critical antigen-presenting cells of the innate immune system are the dendritic cells and macrophages, and they the primary sensors of “danger” signals, and recognize structurally conserved viral proteins via the Toll-like receptors (TLRs- e.g., TLR 3, 4, 7, and 8), which are dominant PRRs recognized by pathogens and are expressed on the surfaces of these immune cells. Defects in TLR function of these innate immune cells have been shown to increase the severity of pneumonia in mice, especially in the context of aging and low-grade chronic inflammation which develops with aging (Inflammaging), and this could be the difference between the aging immune system and disease severity seen during COVID-19 pandemic in the elderly (Zhang and Cao, 2019). Moreover, upon activation, both cell-and stimulus-specific signals are mounted to initiate temporal and spatial responses mediated via both cell-to-cell contact or secretion of interferon (IFN) and tumor necrosis factor (TNF), implying the intrinsic ability of their epigenome to change in real-time following the sensing of danger signal and mounting a vigorous anti-viral host response. This is the way epigenome primes the immunological memory for managing the clear and present danger, and for subsequent future insults.

Thus, to overcome anti-viral restriction by the host, almost all known viruses downregulate the interferon production by immune cells fighting the infection. Interestingly, the subversion of interferons in the host is mainly achieved via the induction of *de novo* methylation of the IFN-γ promoter leading to epigenetic silencing of the interferon secreting genes (ISGs) to block host's anti-viral arsenal (Zhang and Cao, 2019), but the mechanisms independent of epigenetic silencing directly through viral pathogenic mechanisms have also been described for other DNA and RNA viruses to play a role in the silencing of interferon secreting genes (Haller and Weber, 2007).

Lu et al. (2020) have shown the value of a dynamic post-transcriptional RNA modification epigenetic change, known as N6-methyladenosine modification or Adenosine methylation (also known as m6A methylation), in modifying the viral activity and reinstating the host's immune system to fight the virus in a mouse model (Lu et al., 2020). N6-Methyladenosine or m6A accounts for over 80% of all RNA methylation, influencing structure, splicing, localization, translation, stability, turnover, and biology of RNA (Lu et al., 2020). As the m6A exhibits both pro- and anti-viral activities, depending on the virus species and host cell type, its value in disease and treatments is essential. The m6A and m6M affect the viability of specific RNA viruses by modulating viral replication, viral cap structures, innate sensing, and innate immune response pathways (Gonzales-van Horn and Sarnow, 2017). The primary interaction between virus and host during viral infection is affected by m6A, and multiple m6A-modified viral RNAs have been defined, which alter the epi-transcriptome of m6A in the host following viral infection. Viral life cycle right from viral gene expression, replication, and production of progeny virions are all modulated by m6A modifications (Kuppers et al., 2019). It has become evident that m6A methylation makes the virus able to hide from the immune system by masking and mimicking the host RNA to evade immune recognition as being non-self RNA- thereby assuring virus persistence goes undetected in the host. Thus, targeting this viral strategy could pay dividends in anti-viral control.

The SARS-CoV-2 RNA genome has more than 50 potential m6A sites based on the presence of sequence-specific motifs for m6A modification by the RNA methylase complex METTL3/14, including GGACU(T), GGACA, and GGACC. As a result, >0.64% of all adenosines, or 0.18% of all bases, in SARS-CoV-2 RNA could acquire m6A (Kuppers et al., 2019; Lu et al., 2020). Gain or loss of m6A can result in significant functional changes to RNA viruses, at the level of entry, fusion, replication, transmission, host immune evasion, and pathogenesis. Importantly, the m6A epi-transcriptome of host cells, which plays a vital role in resisting viral infection, can also undergo alterations following viral infection (Zaccara et al., 2019; Kim et al., 2020). It is also important to emphasize that as the members of the coronaviruses, including SARS-CoV-2, can encode their own methyltransferases for self-methylating adenosine residues, promoting immune evasion (Zhang and Cao, 2019). Overall, studies on m6A modification in the virus and host can unveil factors that affect SARS-CoV-2 infection and will lead to the identification of novel targets for treatment, and possibly vaccines for COVID-19.

## Specific SARS-CoV-2 Proteins Interfere With Significant Epigenetic Processes of the Host Involving Innate Immunity and Immuno-Pathogenesis

**Figure 3:**
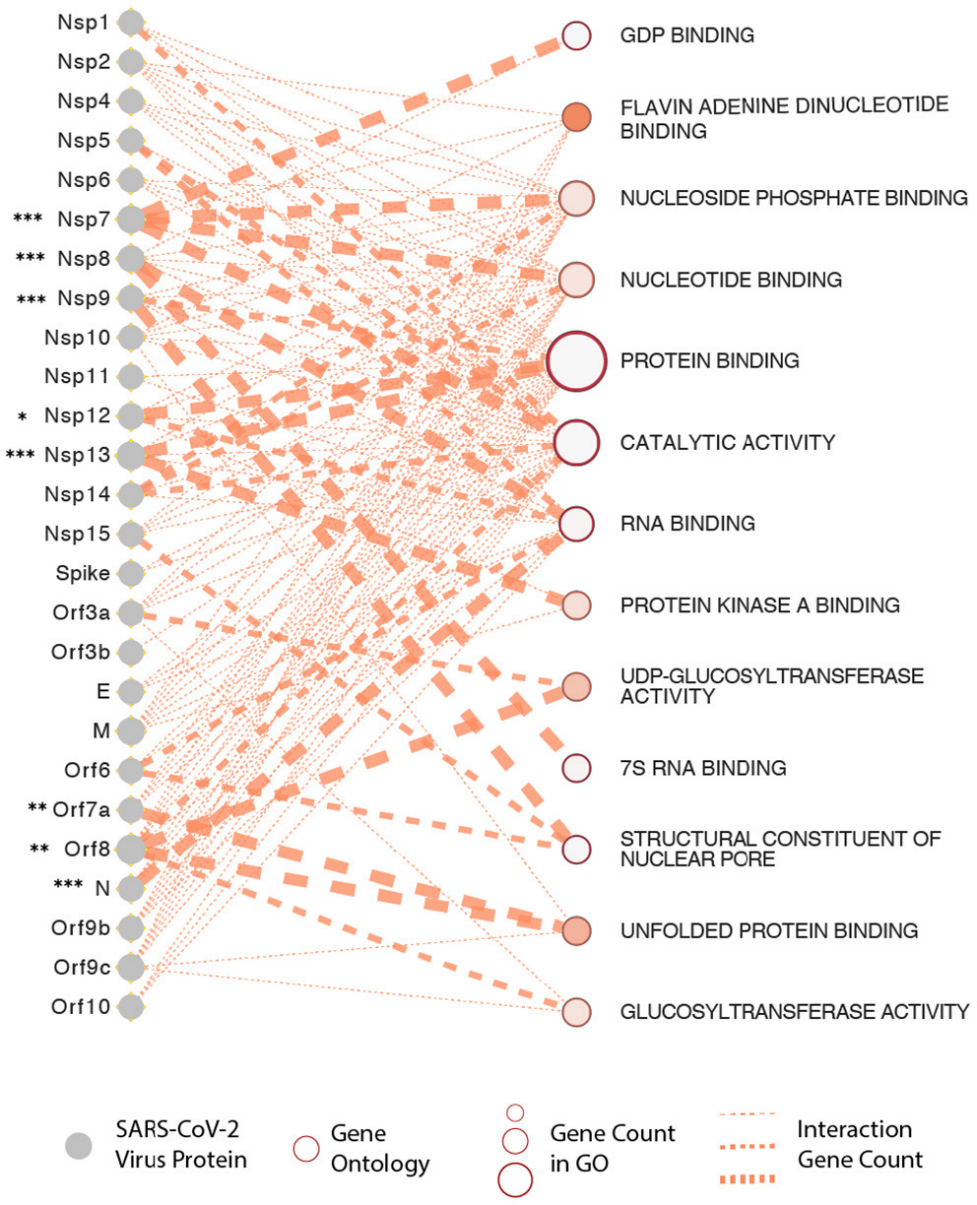
SARS-CoV-2 proteins and gene ontologies of their interacting host genes. Size of gene ontology circle is proportional to the number of genes in the ontology, while thickness of the lines linking SARS-CoV-2 proteins and gene ontologies represents number of interacting genes in the ontology. SARS-CoV-2 proteins interacting with significantly higher number of host genes than expected are marked by asterisks, with representation: *P < 0.05, **P < 0.01, ***P < 0.001.

It is now apparent that all three recently emerged coronaviruses- the SARS-CoV, MERS-CoV, and SARS-CoV-2, have the intrinsic ability to delay pathogen recognition and subjugate, antagonize interferon-stimulated genes (ISGs) effector function. It is thus crucial to understand the role of a variety of SARS-CoV-2-encoded proteins that can effectively and epigenetically modulate host innate immune signaling (Schäfer and Baric, 2017). There are several known viral proteins that associate with viral pathogenesis and are controlled epigenetically (see Figure 3 for an interactome of epigenes that SARS-CoV-2 encodes and its interaction with the host). Below are some of the critical examples of epi-genes in the context of SARS-CoV-2 that have provided some insights into host-virus interactions.

### ORF3b

The SARS-CoV-2, uniquely encodes for a shorter protein-ORF3b (open-reading frame 3b) (Gordon et al., 2020), which has been recently described in hampering innate immune reaction *in vitro* by limiting the induction of the type I interferon response, which is the most crucial aspect of viral pathogenesis. This protein can block host defenses early in the infection cycle by shutting off the host's interferon secretion through an epigenetic mechanism before the T and B cells come into play. SARS-CoV-2 ORF3b is considerably shorter than its SARS-CoV ortholog, encoding a protein just 22 amino acids long due to premature stop codon, compared to the 154 amino acids of SARS-CoV ORF3b. In an *in vitro* experiment, a premature stop codon was introduced into the SARS-CoV ORF3b coding sequence, the resulting 135-amino acid protein was better at suppressing type I interferon than the wildtype SARS-CoV protein was a difference that could be possibly attributed to higher virulence of SARS-CoV-2. Still, these data are preliminary, and further investigation using human samples is needed, because a 56-amino acid variant of SARS-CoV-2-ORF3b circulated in Ecuador showed no evidence of severity, except in some cases (Konno et al., 2020). Moreover, when compared to the related SARS-CoV-1, IFN antagonism could also be attributed to ORF3b, ORF6, and the nucleocapsid (N) gene products (Frieman et al., 2010). In another recent study (Blanco-Melo et al., 2020), this immune response was also shown to be defined by low levels of type I and III interferons juxtaposed to elevated chemokines coupled with high expression of interleukin (IL)-6 suggesting that reduced innate antiviral defenses, coupled with vigorous inflammatory cytokine production, are the most likely drivers of viral pathogenesis. In this context, it is notable that the common respiratory viruses, including influenza A virus (IAV), encode a variety of antagonists to the IFN-I and -III response (García-Sastre, 2017) in comparison to SARS-CoV-2.

### Viroporins

Further, novel coronaviruses (nCoVs) are also known to encode 3 ion-channel proteins, also called Viroporins, such as protein E, ORF3a, and ORF8a. These are small, hydrophobic proteins with a multifunctional ability that aids in modifying cellular membranes and renders permeability with both features assisting the release of virions from infected cells (Carrasco, 1995). They also have additional roles in homeostasis-mediated by protein-protein interactions with host cellular proteins, cellular metabolism, and in enhancing viral growth rates in the host (Gonzalez and Carrasco, 2003). They are particularly common among RNA viruses implicated in causing human diseases, which include HIV, HCV, influenza A virus, poliovirus, respiratory syncytial virus, SARS-CoV, and CoV-2 (Gonzalez and Carrasco, 2003; Nieva et al., 2012; Nieto-Torres et al., 2015; Chen et al., 2019).

Viroporins, via mechanisms that involve intracellular lysosomal disruption and ion-redistribution, lead to the activation of the innate immune signaling receptor NLRP3-(NOD-, LRR-, and pyrin domain-containing 3) inflammasome. This, in turn, triggers the production of inflammatory cytokines [interleukin 1β (IL-1β), IL-6, and TNF] resulting in tissue inflammation, tissue damage, and severe respiratory illness, as seen in COVID-19 pandemic. NLRP3 inflammasome in macrophages can be activated by several viruses and viral proteins, including SARS-CoV-2 (Chen et al., 2019). The inflammasome is a vital arm of innate immunity that detects and senses a variety of endogenous or exogenous, sterile or infectious stimuli encountered within the cell, thereby inducing cellular responses and effector mechanisms to ward off pathogens or threat. Emerging evidence shows epigenetic factors, including DNA methylation and histone acetylation, regulate NLRP3 mRNA expression (Wei et al., 2016), suggesting that NLRP3 inflammasome could be an attractive drug target for SARS-CoV-2 (Shah, 2020). It is worth emphasizing that the transmembrane E protein is another epigenetic player in SARS-CoV-2, which through binding affinity with the BRD2 and BRD4 epigenetic, allows interaction with acetylated histones to regulate gene expression (Lai et al., 2020b). These interactions play a vital role in influencing the inflammatory response and determining the severity of disease in SARS-CoV-2 patients.

### Nsp5

It is the central protease of SARS-CoV-2, and has been shown to interact with one of the epigenetic regulators- the histone deacetylase 2 (HDAC2). This interaction affects inflammatory and interferon responses mediated by HDAC2 (Schäfer and Baric, 2017). In agreement with these findings, Gordon et al. (2020) through a detailed interactome of SARS-CoV-2 and host have also identified high-confidence interaction between Nsp5 and the epigenetic regulator histone deacetylase 2 (HDAC2), and predicted a cleavage site between the HDAC domain and the nuclear localization sequence, suggesting that Nsp5 may inhibit HDAC2 transport into the nucleus potentially impacting the function of HDAC2 in mediating inflammation and interferon response (Chamberlain and Shipston, 2015; Gordon et al., 2020). They also identified an interaction of Nsp5 (C145A) with tRNA methyltransferase 1 (TRMT1), and predict that TRMT1, which can remove its zinc finger and nuclear localization through cleavage by Nsp5, forcing the TRMT1 to localize exclusively in mitochondria.

## Epigenetic Control of ACE2 Dependent Viral Entry, Fusion, Cytokine Storm, Comorbidities and Gender-Related Differences

As discussed in the preceding sections that the spike glycoprotein has two subunits- S1 and S2, where the S1 plays a role in viral attachment to a host cell surface receptor ACE2, and the S2 facilitates the fusion between the virus and the host cell membrane leading to cell-cell fusion involving neighboring cells, eventually leading to the formation of multinucleated giant cells- termed syncytium (Belouzard et al., 2012; Pruimboom, 2020). It is to be emphasized that proper ACE2 functionality is essential for viral entry and local pulmonary homeostasis. The critical step of viral entry comprises a two-step process with S1 glycoprotein engaging ACE2 receptor on the cell surface (Hoffmann et al., 2020) and subsequent cleavage of the S1 by Furin- the second receptor of SARS-CoV-2 (Abassi et al., 2020). Syncytium induction is a common feature unifying all coronaviruses, including SARS-CoV-2 (Mehta et al., 2020; Xia et al., 2020). CpG methylation of host syncytin genes is known (Matoušková et al., 2006) and several viruses make use of the human syncytin genes (encoded by endogenous retroviral elements in the host, which play a vital role in developmental biology) to fuse with the host cell membrane and inducing cell-cell fusion in the tissue sites they infiltrate (Xia et al., 2020).

**Figure 4:**
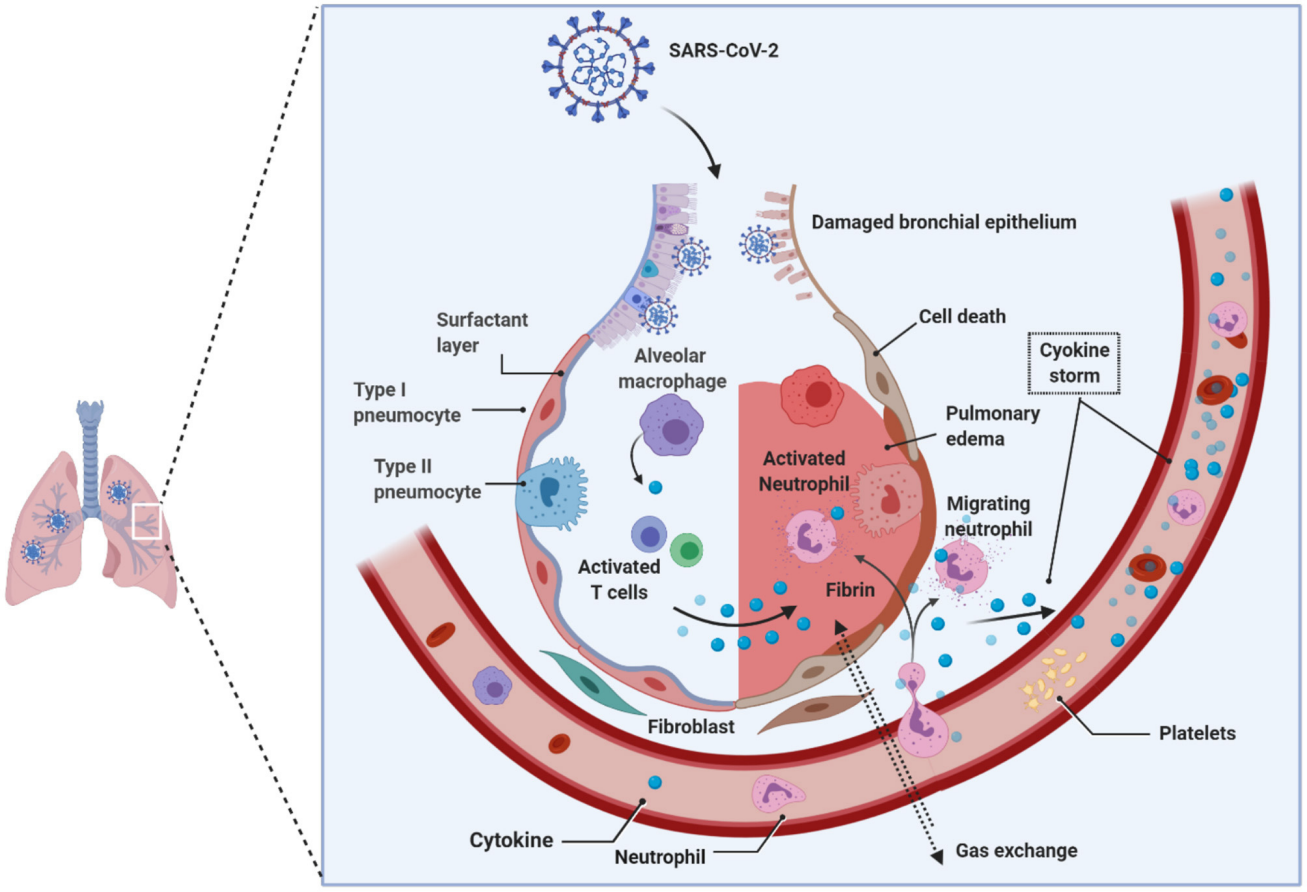
Pathology of lungs in SARS-CoV-2 infection. Early studies on COVID-19 patients biopsies revealed the presence of pulmonary involvement. SARS-CoV-2 infects and use the lung epithelial cells for its propagation. Innate immune system that received the signals from the infection acts as a primary response, such as recruitment of monocytes, macrophages, neutrophils, lymphocytes, and others. Due to the enormous increase in the virions that hijack the immune system response; consequently, hyper activated immune system produces copious amounts of cytokines (i.e., cytokine storm), which damages the host cells. The cumulative effect, caused by the virus and the immune system, leads to the damage of alveoli followed by reduced gas transportation. SARS-CoV-2, Severe acute respiratory syndrome coronavirus 2.

Viruses, such as Epstein Barr-virus, and SARS-CoV-2 can demethylate the host syncytin 1 and 2 genes, resulting in the augmentation of gene transcription (Niller et al., 2014; Pruimboom, 2020). Notable is that the syncytium formation by SARS-CoV-2 is several-fold faster and higher than the SARS-CoV that emerged in 2002, which defines virulence and the propensity to induce cytokine storm in the host seen in acutely sick patients with ARDS during the COVID-19 pandemic (Matsuyama et al., 2020; Xia et al., 2020). The rapid and massive secretion of inflammatory cytokines and chemokines is the unique feature of SARS-CoV-2 patients with ARDS. Huang and colleagues showed that notably higher levels of IL-2, IL-7, IL-10, G-SCF, IP10, MCP1, MIP1A, and TNFα were found uniquely in the peripheral blood of patients admitted to the intensive care unit (ICU), suggesting that the cytokine storm is strongly associated with the disease severity in SARS-CoV-2-infected patients, which could be attribute to apoptosis and subsequent cell death due to syncytium formation (Huang et al., 2020) (Figure 4). Surprisingly, the vast majority of ARDS patients in the ICU are males during the continuing COVID-19 pandemic, and the reasons for this disparity, still, remain unclear.

To explain the reasons for this disparity, it is essential to know the homeostatic balance between angiotensin I-converting enzyme (ACE1) and ACE2. The ACE1 and its homolog ACE2 (Tipnis et al., 2000) are two antagonist enzymes of the RAS pathway that together maintain homeostasis by counterbalancing each other (Burrell et al., 2004). Any imbalance between ACE1 and ACE2 tilts the balance between the healthy state and disease severity, and it has been shown that this imbalance between ACE1 and ACE2, mediated by SARS-CoV-2-mediated suppression of ACE2, results in RAS over-activation and pulmonary shut-down which is a consequence of induced- pulmonary edema, inflammation, hyper-proliferation, and cytokine storm together causing ARDS (Figure 4). It is important to reiterate that the manifestation of ARDS has differed between males and females during the COVID-19 pandemic (Gemmati et al., 2020). Despite ACE1 I-allele being overrepresented among females, the D-allele (associated with a high level of ACE1) is expressed at higher levels among males, suggesting higher preponderance of ACE1/ACE2 imbalance among males resulting in disease severity (Hoffmann et al., 2020; Zhang et al., 2020). It has also been suggested that females might have a higher degree of heterodimer assembling than males, which might show the different receptor-binding affinity of SARS-CoV-2 spike protein. To support these observations, it is known that the ACE2 (locus Xp22.2) is located on the X-chromosome, and according to Gemmati et al. (2020), the X-linked heterozygous alleles could give females a significant advantage and a greater sexual dimorphism in counteracting viral infection and less severe manifestation of respiratory events as seen during COVID-19 pandemic in comparison to their male counterparts (Gemmati et al., 2020).

Now that we know the ACE2 expression in the lungs is associated with enhanced susceptibility to infection and its severity (Gurwitz, 2020; Leung et al., 2020), it is important to understand how the epigenetic mechanisms control the susceptibility to SARS-CoV-2 infection in concert with age and gender. Recently, Pinto et al. (2020) have demonstrated that ACE2 gene expression and enzyme production are also highly enriched in the host lung epithelium (Zill et al., 2012), is under tight epigenetic control. This epigenetic control, based on methylation patterns of diverse but specific CpG islands in the ACE2 gene, is associated with age and gender (Corley and Ndhlovu, 2020).

What is more tantalizing is that while the ACE2 is expressed in diverse tissues and organs (showing the highest expression in heart, kidney, small intestine, testis, adipose tissue and thyroid; intermediate level expression in lungs, liver, bladder, colon, and adrenal gland; and the lowest expression in the bone marrow, spleen, brain, muscles, blood, and blood vessels), its methylation rate in the lung epithelial cells is lower in comparison to other tissues, further implying the highest transcription and expression rate of ACE2 (Corley and Ndhlovu, 2020). Because both age and gender correlate with hypomethylation of the ACE2 gene in the lung tissue, it may have the potential to provide a possible explanation for age-and gender-related mortality seen with SARS-CoV-2 infection during COVID-19 pandemic, especially in elderly men and smokers characterized by hypomethylation of ACE2-and-interferon genes (Pinto et al., 2020). In contrast women, children, and non-smokers display DNA hypermethylation and lower expression of ACE2 and interferon proteins, and as a consequence show better protection to SARS-CoV-2 as seen during the COVID-19 pandemic (Holmes et al., 2019; Pinto et al., 2020), which is in full concordance with the process of epigenetic aging (Jones et al., 2015).

Further, as many comorbidities associated with SARS-CoV-2 infection get unraveled, it is becoming clear that the mechanism across comorbidities generally involves severe lung infection, respiratory distress, and cytokine storm followed by mortality. Recently, epigenetic dysregulation via hypomethylation of ACE2 coupled with demethylation of interferon and cytokine-regulated genes, and enhanced NF-kB (nuclear factor kappa light chain enhancer of activated B cells) have been shown to contribute to disease severity in SARS-CoV-2-infected patients with lupus (Sawalha et al., 2020). To explain this better, oxidative stress induced by viral infections exacerbates DNA methylation defect in lupus leading to ACE2 hypomethylation in concomitance with high viremia. Moreover, the demethylation of interferon-regulated genes, such as NFκB and cytokine genes, exacerbate the immune response taking it into a hyper-drive mode resulting in a cytokine storm (Sawalha et al., 2020). This agrees further with the significant role epigenetic dysregulation plays in viral entry, development of viremia, hyper-immune response to SARS-CoV-2, inflammatory process of cytokine storm, and, consequently, disease severity. These observations suggest that specific subgroups of patients, with possibly unfavorable epigenetic profiles, could be more susceptible to SARS-CoV-2 than others who do not have this profile. This has been evident from the higher global mortality rates mainly among SARS-CoV-2-infected individuals who displayed comorbidities such as age, autoimmune disorders, obesity, diabetes, cardiovascular ailments (hypertension), smoking, and immunocompromised cancer patients, and others (Mehta et al., 2020). This mortality, across different comorbidities, as we have seen during COVID-19 pandemic, could be attributed to high expression of the lung ACE2 protein and hypomethylation of its gene, coupled with significant demethylation of interferon genes prompting respiratory distress seen more frequently during COVID-19 pandemic (Sawalha et al., 2020).

Apart from receptor variability defining the outcome of SARS-CoV-2 disease, other host variabilities that may associate with mortality and survival with infection remain unaccounted for, and it is vital to consider interhost variability along with what has been discussed herein. For instance, an important consideration should be given to major-histocompatibility-complex antigen loci (HLA), genetic variability in cytokine and interleukin genes (IL-1, IL-6, and TNFs) which regulate the immune and inflammatory response, along with other unknown host and viral factors that may serve as prototypical candidates in defining genetic susceptibility to severe SARS-CoV-2-infection (Chan et al., 2020). To understand these, genomic visualization of differentially expressed functional genetic variants and epigenetic profiles of methylation and small non-coding RNAs will clarify these associations.

## Epigenetic Modulation of SARS-CoV-2 Infection and Its Predilection With Age

One of the most critical features defining the COVID-19 pandemic should be the mortality rates among the elderly population; therefore, it is essential to examine and discuss in brief the underlying mechanistic and epigenetic associations of SARS-CoV-2 with age and find out why age is such a significant comorbidity. As has been seen across different continents, the virus is highly contagious, especially for elderly individuals, attributed not only to a higher rate of mortality but also to a higher proportion of infected cases (Cortis, 2020; Mueller et al., 2020).

The changes in gene expression, accompanied by the epigenome's dysregulation during the aging process, have been analyzed by several studies. The theory of aging postulates, which suggests lifetime accumulation of epigenetic changes as a consequence of the loss of resilience (Oberdoerffer et al., 2008), are driven in part by the redistribution of chromatin modifiers (nuclear proteins sirtuins [SIRT1/6/7], HDAC1 and polyadenosine diphosphate-ribose polymerase-1 [PARP1]) to sites where the dsDNA break repair occurs, resulting in epigenetic “noise” which conceals cellular identity (Oberdoerffer et al., 2008; Dobbin et al., 2013; Gorbunova and Seluanov, 2016). Interestingly, the viral Nsp5 protein (an epigenetic regulator) interacts with HDAC1 (Gordon et al., 2020). The whole process, which manifests in the form of DNA methylation, sets the pace of the biological clock in hematopoietic cells (Horvath, 2013). Such age-related changes negatively impinge on the immune system, adaptive and innate immune and antiviral defenses, and immune memory dysfunction (Schäfer and Baric, 2017; Keenan and Allan, 2019). Coronaviruses mediate epigenetic changes, possibly by accelerating the rate of aging of the immune system, antagonizing host antigen presentation by altering DNA methylation, and silencing the very genes that encode the major histocompatibility complexes (Menachery et al., 2018). It is now becoming clearer that together these epigenetic insults can delay interferon response gene activation, coupled with epigenetic changes to histone methylation and, also to the long non-coding RNAs (Menachery et al., 2014).

ACE2 is regulated in the body transcriptionally (post-transcriptionally-and-translationally) (Patel et al., 2016), and this is controlled by methylation. Significantly, methylation at one of seven CpGs in the ACE2 promoter is known to decrease with age, which is further supported by studies on both mice and rats where ACE2 expression decreased with age and was associated with aortic fibrosis and inflammation (Xudong et al., 2006).

Other explanations are the sirtuins (SIRT1-7), which are a family of NAD^+^-dependent lysine deacetylases and are known to play a role in controlling stress resistance and maintain defenses against pathogens (Kwon et al., 2008). It is also essential to note that SIRT1 is a nuclear histone deacetylase and plays a vital role in suppressing viral replication and subsequent inflammatory events (Kwon et al., 2008). Depletion of NAD^+^, particularly during aging and also during SARS-CoV-2, is attributed to its increased NAD^+^ consumption by the CD38^+^ glycohydrolase (Chini et al., 2018), and by viral ADP ribosylhydrolase in humans infected with SARS-CoV-2 (Blanco-Melo., 2020) leading to disruption of processes guiding cell-signaling, DNA repair, gene regulation and apoptosis (Kwon et al., 2008). This suggests that age-related decline in NAD^+^ coupled with the mis-localization of SIRT1 and 6 spanned across the genome during aging may be one of the primary underlying reasons for SARS-CoV-2 predilection to age leading to severe disease manifestation seen globally among the elderly population during the COVID-19 pandemic. Lastly, glycosylation changes may predispose aged individuals to SARS-CoV-2 infection and disease severity (Nicholls et al., 2007), as glycosylation patterns also change with age.

Recently, Corley et al. (2021) examined genome-wide DNA methylation (DNAm) profiles of peripheral blood mononuclear cells from 9 terminally-ill, critical COVID-19 patients confirmed lymphopenia in severe COVID-19 and revealed a high percentage of estimated neutrophils suggesting perturbations to DNAm associated with granulopoiesis. A DNAm signature of severe COVID-19 characterized by hypermethylation of IFN-related genes and hypomethylation of inflammatory genes was observed in their studies. Further, the epigenetic clock analyses revealed the severe SARS-CoV-2 infection to be associated with an increased DNAm age and elevated mortality risk according to GrimAge, further demonstrating the epigenetic clock as a predictor of disease and mortality risk, which has not been shown before. The epigenetic DNAm signature of severe COVID-19 in blood could potentially serve as a useful biomarker in clinical assessments, predicting pathogenicity, and also in designing new therapeutic targets against SARS-CoV-2.

## Non-Coding RNAs- (Epi-Transcriptome) and SARS-CoV-2 Infection

The non-coding RNAs (ncRNAs), such as tRNAs, miRNA, ribosomal RNA (rRNAs), and spliceosomal RNAs, together make the ‘epi-transcriptome’ which is a layer that influences epigenetic regulation at the RNA level (Peng et al., 2010; Baldassarre et al., 2020). ncRNAs are suggested to regulate the host response, including innate immunity, as indicated by the widespread differential regulation of long ncRNAs in response to viral infection. Therefore, it is difficult to compile all the individual ncRNA in terms of their significance in SARS-CoV pathogenesis because not much is known in the context of SARS-CoV-2 pathogenesis, and that most studies done to date have been in mouse models. Due to this, and also to facilitate some understanding, we have specifically chosen the only miRNA as there are current studies to explain their possible role in SARS-CoV-2 pathogenesis using human and viral miRNA data (Khan et al., 2020).

MicroRNAs are small ncRNA molecules and are the master regulators of gene expression. Many viruses are known to utilize the host gene machinery to subvert this regulatory process. In this context, it is essential to note that the subversion occurs through the viral miRNAs, which mimic host-miRNAs and regulate host gene expression (Lai et al., 2014). Because miRNA can epigenetically control antiviral mechanisms by stimulating the innate and adaptive immune systems (Trobaugh and Klimstra, 2017), the virus targets this epigenetic regulator and subvert it to guide viral propagation by modulating cellular pathways without being detected by the host immune response (Głobińska et al., 2014). Interference of viral miRNA with the host miRNA is pinned into disrupting host-immune defenses and antiviral immunity, known collectively for ncRNA (Peng et al., 2010). For instance, the nucleocapsid protein of coronavirus OC43 binds to miR-9 and activates NF-κB to play a vital role in innate signaling (Lai et al., 2014).

A study by Khan et al., which uses machine learning and knowledge-based approaches, has elegantly shown various facets of the host-virus interplay at the molecular level between host miRNAs and SARS-CoV-2 (Khan et al., 2020). These miRNAs are subverted by the virus and deregulate major antiviral immune signaling pathways, in addition to abnormal regulation of several other host pathways, thereby promoting viral pathogenesis. Identification of several host antiviral miRNAs that can target the SARS viruses, and also SARS viruses' encoded miRNAs targeting host genes has provided a powerful visualization of host-virus interaction and putative functional entities. The demonstration that SARS-CoV and SARS-CoV-2 viral miRNAs could target broad immune-signaling pathways, but only some of the SARS-CoV-2-encoded miRNAs uniquely and selectively targeted immune signaling pathways like- autophagy, IFN-I signaling, and others are important. This suggests distinct immune-escape mechanisms for SARS-CoV-2, coupled with prolonged latency in their hosts without displaying any symptoms, which continues to be a feature of viral transmission from the asymptomatic and pre-symptomatic individuals in the COVID-19 pandemic. Further, through these bioinformatic analyses, the data also shows that SARS-CoV-2 can modulate some key cellular pathways leading to increased anomalies in patients with comorbidities like- cardiovascular diseases, diabetes, breathing complications, and others.

Together, these data suggest that miRNAs are master epigenetic regulators.

Four host miRNAs (hsa-miR-654-5p, hsa-miR-198, hsa-miR-622, hsa-miR-323a-5p) for SARS-CoV and 3 miRNAs for SARS-CoV-2 (hsa-miR-17-5p, hsa-miR-20b-5p, hsa-miR-323a-5p) were identified against, which have potential antiviral roles during infections. Overall, the study by Khan et al. was able to dissect several interesting functional and highly relevant facets of miRNA regulation in SARS-CoV-2 infection including that-

1. Infection-induced host miRNAs can function as a pro-viral factor by inhibiting host immune surveillance pathways.
2. Host miRNAs targeting SARS-CoV and SARS-CoV-2 can play crucial roles in neutralizing the virus, and
3. Host miRNAs targeted by SARS-CoV-2 downregulated pathways related to the comorbidities and are highly pertinent to the COVID-19 pandemic.

Notable is that the observations presented in the study by Khan et al. (2020) functionally correlate with the protein-protein interactome study by Gordon et al. (2020). If these two studies can integrate the dataset, as they are from human origin, a finer network of protein-miRNA regulatory interactome can be obtained, which will greatly facilitate experimental validation and discovery of specific drug and vaccine targets. Additionally, it will be interesting to know how these miRNAs interact with the virally-encoded epigenes.

## Microbiome- an Unrecognized Layer of the Epigenome

### Microbiome in Nasal-and Oropharyngeal Cavities- Role in Influenza and SARS-CoV2 Infections

We already know about the vital role gut microbiome plays in various infections. While it is known that a healthy intestinal flora is closely related to the maintenance of pulmonary and systemic health by regulating the host immune homeostasis, the role of the “gut-lung axis” has also been widely recognized (He et al., 2020). Furthermore, the prevention and treatment strategies for SARS-CoV-2 infection, taking into account the intestinal microbiota, are receiving considerable attention (Gou et al., 2020; Li et al., 2020b). While it is beyond the scope of this review to discuss both the gut and nasal microbiome, this review focuses on the microbiome of the nasal-and oropharyngeal cavities as they are the most important and the first ports of viral entry, in addition to being the most affected ones during SARS-CoV-2 infection. It also makes perfect sense because the SARS-CoV-2 entry factors and innate immune genes are highly expressed in nasal epithelial cells, according to a recent study (Sungnak et al., 2020), highlighting the potential role of these cells in initial viral infection, spread, and clearance. Thus, there are opportunities within for controlling, diagnosing, and treating viral infections, including SARS-CoV-2.

A meta-transcriptomic study of 187 individuals (62 with SARS-CoV-2 infection and 125 with non-SARS-CoV-2-related pneumonia) (Corley et al., 2021) constructed a database comprising of 18,556 species of bacteria, viruses, fungi, and parasites, and analyzed the meta-transcriptomic sequencing data from the patient cohort. Through the analysis of microbiome and host responses, the authors built a host gene classifier based on the host transcriptional patterns that can prove valuable in predicting disease severity. Surprisingly, the airway microbiota of SARS-CoV-2-infected patients showed reduced alpha diversity, suggesting dysbiosis, which was characterized by over 18 taxa that showed a differential abundance of microbial species, along with the detection of pathogenic microbes in about 47 percent of SARS-CoV-2-infected patients, with 58 percent of these being the respiratory viruses, in addition to Candida albicans and human alphaherpesvirus-1 (commonly known as herpes simplex virus-1) detected as opportunistic pathogens. Notably, the higher opportunistic infection was with viruses than with bacteria or fungi in SARS-CoV-2 patients implying the importance of co-pathogens playing a significant role in disease severity.

The parallel host gene analysis in this study revealed the most prominent transcriptional signatures of genes primarily associated with immune pathways such as cytokine signaling, which has been commonly seen with most respiratory viruses. The cytokine signaling was the most significantly deregulated, followed by the innate immune system and neutrophil degranulation pathways. This suggests a vital role of innate immunity and cytokine signaling in SARS-CoV-2 infection, which has been discussed extensively in various preceding sections of this review. It is too early to say how metagenomic analysis can be integrated into classifying disease severity and diagnostic modules, but these studies have already shown that promise.

## What Does Virus-Host Interactome of Protein-Protein Interactions Tell Us About Epigenetic Control of SARS-CoV-2?

On the one hand, viruses activate immune gene expression leading to the induction of host immune response, and, on the other hand, they can also hyper-methylate immune genes, thereby silencing the host immune response. Notable is that viruses do not directly methylate these host resistant areas by themselves, but they achieve this by recruiting host's immune proteins to do their dirty work and methylate these regions to make subversion of the human gene machinery their own benefit (Kuss-Duerkop et al., 2018). Therefore, it is crucial to understand the protein-protein interaction map between the virus (SARS-CoV-2) and the host, in particular, the proteins that carry-out epigenetic gene expression regulation. A holistic understanding of the process and the intrinsic associations between host and viral proteins is of paramount importance concerning developing effective vaccines, finding new druggable targets, and re-purposing the pre-existing drugs.

Gordon et al., in one of the most comprehensive analysis, cloned, tagged and expressed 26 of the 29 viral proteins in human cells and identified the human proteins physically associated with each other using affinity purification mass spectrometry (AP-MS), which further identified 332 high confidence SARS-CoV-2-human protein-protein interactions (PPIs) (Gordon et al., 2020). The first detailed virus-host interactome has defined each viral protein and annotated individually to functional entities- such as DNA replication (Nsp1), epigenetic and gene expression regulators (Nsp5, Nsp8, Nsp13, E), vesicle trafficking (Nsp6, Nsp7, Nsp10, Nsp13, Nsp15, Orf3a, E, Orf8), lipid modification (Spike), RNA processing and regulation (Nsp8, N), ubiquitin ligases (Orf10), signaling (Nsp8, Nsp13, N, Orf9b), nuclear transport machinery (Nsp9, Nsp15, Orf6), cytoskeleton (Nsp1, Nsp13), mitochondria (Nsp4, Nsp8, Orf9c), and extracellular matrix (Nsp9).

In addition to identifying novel protein-protein interactions, their study also identified four viral epigenetic proteins- Nsp5, Nsp8, Nsp13, and E, respectively (Gordon et al., 2020). Several cellular proteins implicated in innate immune signaling targeted by several SARS-CoV-2 viral proteins were also identified. For instance, Nsp13 showed interaction with two key players of the IFN signaling pathway, including TANK-binding kinase 1 (TBK1) and TANK-binding kinase 1-binding protein 1 (TBKBP1/SINTBAD). Further, Nsp13 interacts with the TLE family's multiple proteins, which are known to modulate the NF-κB inflammatory response (Ramasamy et al., 2016). Orf6 of SARS-CoV has been shown to antagonize host interferon signaling (Frieman et al., 2007); and this study identified a high-confidence interaction between SARS-CoV-2 Orf6 and NUP98-RAE1, an interferon-inducible mRNA nuclear export complex that is hijacked or degraded by multiple viruses, and which acts as a restriction factor for Influenza-A infection (Timms et al., 2019). One of the more significant interactions identified was the transmembrane protein E binding to the bromodomain-containing proteins BRD2 and BRD4 potentially disrupting BRD-histone binding by mimicking histone structure, suggesting that protein E mimics the histone to disrupt its interaction with BRD2, thus inducing changes in host's protein expression that are beneficial to the virus (Gordon et al., 2020). Therefore, a large number of these interactions are pinned into subverting and hijacking host antiviral defense machinery and immune evasion.

To understand this better, we have constructed a gene ontology (Figure 3) taking the protein data from the study by Gordon et al. We show that the SARS-CoV-2 epigenetic proteins are predominantly networked with various RNA-and-protein binding activities (protein binding, RNA binding, Flavin adenine dinucleotide binding, nucleotide binding, protein kinase binding, unfolded protein binding, and nucleotide phosphate-binding) (Gordon et al., 2020). Furthermore, when all the viral proteins were visualized together for interactome, protein, and RNA binding also emerged at the core of virus-host interaction (Figure 3). In support of the SARS-CoV-2 interactome and to solidify in a practical sense, it is already known that the plus-strand (+) RNA viruses co-opt host RNA-binding proteins (RBPs) for its own functional benefit, which includes viral replication. The RBPs affect the recruitment of viral (+) RNAs for replication, while other proteins subvert host RBPs, thereby assisting the assembly of the membrane-bound replicase complexes, and regulating the activity of replicases in controlling + or -strand RNA synthesis (Li and Nagy, 2011).

## Future of Epigenetics-Based Treatments for Viral Infections

Although several non-epigenetic drugs have been re-purposed for treating SARS-CoV-2 (summarized in Figure 2 and reviewed in Saksena, 2020), none of the drugs, which include Remdesevir, Lopinavir, Favipiravir, and Ribavarin, and Hydroxychloroquine targeting viral translation, transcription, protease, and autophagy, have indeed proven to be fully effective and have mixed results in suppressing the virus despite targeting the most relevant checkpoints in the SARS-CoV-2 viral life cycle (Figure 2) (Bonam et al., 2020a). It is likely that we have misread the virus from the prior knowledge on other SARS viruses or that the SARS-CoV-2 is a new entity and, the drugs should be specifically developed for. Alternatively, it may be that a combination of several pre-existing or new drugs should be considered in treating SARS-CoV-2.

Other approaches that may hold considerable promise are immunotherapies, monoclonal antibodies, and convalescent plasma (CP), where the CP derived from infected recovered individuals has shown the best results in terms of recovery (Duan et al., 2020). Several immunotherapies are under clinical trials (Bonam et al., 2020b, 2021), and they have yet to declare results against SARS-CoV-2. In regards to vaccines, there are several at different stages of clinical trials (DNA vaccine, live attenuated, and protein epitope-based vaccines, viral vector-based vaccine, RNA vaccines, but the vaccines whose efficacy has been proven in Phase 3 clinical trials and have obtained emergency use authorization for human use by the Food and Drug Administration (FDA) are mRNA-1273 by Moderna TX Inc.; mRNA- BNT162b2 by BioINTech/Pfizer Inc.; Oxford–AstraZeneca chimpanzee adenovirus vectored vaccine ChAdOx1 nCoV-19 (formerly AZD1222); and J&J adenovirus vector-based JNJ-78436735 vaccines. These are the vaccines that have been rolled-out globally for mass vaccination. Other vaccines that have not been approved by the FDA, but have been rolled-out for human use through specific country-based approval are the Chinese Vaccine (SinoVac) by Sinopharm and the Russian Vaccine (Sputnik) (https://www.cdc.gov/coronavirus/2019-ncov/vaccines/different-vaccines.html).

Although there are no current epigenetic drugs, several strategies are being considered that have a promising future, are discussed below.

### Histone Modifying Approaches in SARS-CoV-2 Treatment

Respiratory viruses, including influenza and coronaviruses, have an incessant need for modifying histone proteins for their own advantage. The tactics they employ to subjugate human epigenetic machinery are by histone mimicry, where viral proteins replace a part of the host's epigenetic machinery, leading to gene silencing and enhanced inflammatory response (Schaefer et al., 2013). Thus, these imposter proteins can be excellent targets for subduing viral infection from incurring more insults on the host's innate immune system. It is to be noted that not all viruses use histone mimicry, so we cannot have a generalized epigenetic drug.

### Probiotics as Immunostimulants

It is believed that a profound understanding of microbiome in different local areas of the host can explain how microbiome approaches could be implemented in future therapeutics to treat viral infections, including SARS-CoV-2. There is a growing body of evidence that the human microbiome is closely tied to human innate and adaptive immunity. The human pharyngeal microbiome residing at the juncture of digestive and respiratory tracts plays a vital role in preventing respiratory tract infections. It is important to reiterate that respiratory distress or ARDS is essential to the main feature of SARS-CoV-2 disease. Pharyngeal microbiome seamlessly interacts with the local epithelial and immune cells, and together, they form a unique micro-ecological system that is fully capable of evoking host immune responses to eliminate invading viruses (Kumpitsch et al., 2019).

While most microbiome-manipulations or probiotic studies have focused on gastrointestinal diseases, there is a growing need for looking at the local area microbiome, which may have considerable relevance in respiratory viral infections. There is evidence that the nose and throat's microbiome community structure before infection with a virus is associated with susceptibility to influenza (To et al., 2020b). If one or more stable microbiomes are found that inhibit SARS-CoV-2, it can reduce dependence on strain-specific vaccines.

Studies in a mouse model demonstrate that a probiotic nasal spray reduced signs of infection and virus titers while having a positive effect on weight change and survival (Harata et al., 2010). Many studies have focused on different strains of Lactobacillus against H1N1, vesicular stomatitis virus, and pneumonia virus of mice (PVM), an ssRNA virus. Moreover, nasal probiotics have also been shown to induce the expression of viral defense genes such as IFN-beta, IL-12, and IL-10. They can also regulate the TRL7 signaling pathway, which is governed by the microbiota against respiratory tract influenza A viral infection (Harata et al., 2010). Given the success of these studies, it will be productive to test different bacterial species as probiotics against SARS-CoV-2. The causal mechanisms of protection that have been explored in these animal studies fall along two lines: direct probiotic-viral interactions, and immunomodulation due to the probiotic. Supporting this, there is evidence *in vitro* that Lactobacilli can bind and inhibit strains of vesicular stomatitis virus in a cell culture model (Botić et al., 2007). Probiotics also secrete a range of antiviral metabolites, such as violacein that can kill viruses or inhibit replication. As such, members of the microbiome could reduce initial viral titres in the airways and, therefore, can lead to a milder infection (Channappanavar and Perlman, 2017). Overall, the prevention of a cytokine storm may be vital in the treatment of COVID-19 severe diseases using microbiome that is found in uninfected individuals.

### Attacking Virus Through the Awakening of Innate Immunity Sensors

While the adaptive immunity and creation of a vaccine will undoubtedly help the pandemic globally, but harnessing the host innate immunity combat COVID-19 has immense therapeutic potential. Immunomodulation through activation of toll-like receptor 5 (TLR5), which are involved in innate immunity sensing, can be an innovative approach to combat SARS-CoV-2. This is because the TLR5 recognizes the structural protein of the flagellum in motile Gram-positive and Gram-negative bacteria (Hayashi et al., 2001), and it has been hypothesized that flagellin, which could act as a trojan horse “danger” signal by favorably tricking the host for a need to subdue bacterial infection but instead triggering antiviral response against the SARS-CoV-2. Similar approaches are being trialed for influenza viruses (Golonka et al., 2020). As discussed in the preceding sections of this review, the m6A methylation approach could also lead to the first epi-vaccine, which targets the innate and adaptive immune responses (Lu et al., 2020).

## Conclusions

This review has provided an exhaustive overview of the most recent development in the field of SARS-CoV-2 epigenetics to initiate a discussion among scientists and clinicians so that future disease management can be improved by taking various strategies and approaches discussed herein. It is important to reiterate that we need to have approaches in place that can prevent the spread of infection and the successful treatment of infected individuals. These approaches should be around genomic mapping, surveillance, prediction, identifying disease subtypes, proteomic mapping to define smarter strategies, identifying protein-protein interaction to map host-virus interactions, future vaccines, and drug candidates and identifying small RNA molecules that provide durable epi-antiviral control. Moreover, the identification of more existing drugs that can be re-purposed should be made with urgency, as no new drug formulations have passed FDA approval. There is a dire need for an open international collaboration to address the problem through global partnerships between multinational pharma-companies, mainly in vaccine and drug development, as they have resources and ready infrastructure. To derive any drug or a vaccine, the knowledge of viral genome mapping from a global perspective is essential. Having viral sequences at hand will help both clinicians and scientists monitor hotspots, develop new prognostics/diagnostics, facilitate vaccine development, and predict viral genetic changes with host-specific tropism. This genomic knowledge of small RNA, methylation, chromatin structure modulation, and microbiome, will also facilitate the understanding the role epigenetics plays in viral infection. Although there are no epigenetic drugs to treat SARS-CoV-2 disease, epigenetics-based drugs have a bright future in curing infections. The approaches discussed herein will stimulate ideas before a complete understanding of epigenomic landscapes is achieved. Furthermore, it is not clear whether the some of these vaccines are safe in the elderly, as seen in Norway with the use of the Pfizer-BioINTech vaccine which led to the death of 30 elderly people (https://www.tga.gov.au/media-release/norwegian-investigation-covid-19-vaccination-risks-elderly), but the vaccination of the elderly and younger-age categories has been successful in general with all 4 approved vaccines. It remains to be seen how the long-term functional immunity develops in the vaccinees and, how long the adequate protection against SARS-CoV-2 will last? Nonetheless, long-term favorable and adverse consequences of COVID-19 vaccines remains to be elucidated.

## Methods

Taking the data from Gordon et al. (2020), we were most interested in the viral genes that interfere with host epigenetic regulation. We used a Web based tool g:Profiler (Reimand et al., 2016) to perform gene ontology analysis of the host genes interacting with SARS-CoV-2 proteins. Significantly enriched Molecular Function gene ontologies and their interacting SARS-CoV-2 proteins were visualized by Enrichment map plugin (Merico et al., 2010) in Cytoscape (Shannon et al., 2003). Hypergeometric test was used to test for SARS-CoV-2 proteins that are interacting with a significantly higher number of host genes in the ontologies than expected.

## Author Contributions

NS conceptualized the review and wrote it in consultation with MM-S and SB. SB contributed to various sections on therapy and the figures. NS contributed to several sections on epigenetics and viral infection. All authors contributed to the article and approved the submitted version.

## Conflict of Interest

NS is employed by the company Epigenes Australia Pty Ltd. Melbourne, Australia.

The remaining authors declare that the research was conducted in the absence of any commercial or financial relationships that could be construed as a potential conflict of interest.
